# Experiences of Home-Based Participation in a Digitally Distributed Yoga Intervention in Breast Cancer Rehabilitation: Qualitative Content Analysis

**DOI:** 10.2196/75975

**Published:** 2025-11-20

**Authors:** Maria Birgitta Nilsson, Mialinn Arvidsson Lindvall, Per Wilhelm Tage Fessé, Susanne Hellerstedt-Börjesson, Emma Ohlsson-Nevo, Anna Duberg

**Affiliations:** 1 University Health Care Research Centre Faculty of Medicine and Health Örebro University Örebro Sweden; 2 School of Health, Care and Social Welfare Mälardalen University Västerås Sweden; 3 Centre for Research and Development Uppsala University/Region Gävleborg Gävle Sweden; 4 Institute of Health and Care Sciences Sahlgrenska Academy University of Gothenburg Gothenburg Sweden; 5 Lifestyle and Rehabilitation in Long-Term Illness Department of Public Health and Caring Sciences Uppsala University Uppsala Sweden; 6 Centre for Clinical Research Dalarna Uppsala University Falun, Uppsala Sweden; 7 Department of Surgery Faculty of Medicine and Health Örebro University Örebro Sweden

**Keywords:** breast cancer, yoga, telerehabilitation, cancer rehabilitation, cancer-related fatigue, empowerment, self-care, mental health, eHealth

## Abstract

**Background:**

Breast cancer is the most diagnosed cancer among women, with survivors often experiencing long-term symptoms such as cancer-related fatigue, which significantly impacts quality of life. Yoga has demonstrated potential in alleviating cancer-related fatigue and enhancing overall health and quality of life. Digital interventions are increasingly recognized as a feasible approach to cancer rehabilitation. However, digitally distributed home-based yoga interventions in breast cancer rehabilitation remain underexplored. More research in this area is essential to achieve a deeper understanding of participants’ experiences.

**Objective:**

This study aims to explore women’s experiences of participating in a digitally distributed, home-based yoga intervention in breast cancer rehabilitation.

**Methods:**

This qualitative study used an inductive content analysis approach. Semistructured interviews were conducted with 20 women who had undergone breast cancer surgery and participated in a 12-week digitally distributed, home-based yoga intervention as part of the randomized controlled multicenter trial Digital Yoga Intervention in Cancer Rehabilitation (DigiYoga CaRe). The intervention consisted of 2 home-based yoga sessions each week: 1 live-streamed group class led by an experienced yoga instructor and 1 prerecorded self-paced practice video. Interviews were conducted within 1 month of completing the intervention, using a semistructured guide with 12 open-ended questions covering physical and mental experiences, daily life impacts, and reflections on the digital format (live-streamed and prerecorded video). The interview transcripts were analyzed using conventional content analysis to identify subcategories and categories, providing insights into participants’ experiences.

**Results:**

The interviewees reported that the home-based yoga intervention helped them to actively manage their illness and treatment-related side effects, promoting mental recovery and relaxation, physical function and activity, and self-care practice and routines. They commented that it fostered resilience and empowerment through increased body awareness and by regaining trust in their bodies, self-confidence, and hope for recovery. It was described as a shift in focus toward progress and well-being, equipping them with new coping strategies for their daily lives. They perceived the telerehabilitation format as a safe and user-friendly rehabilitation option, providing support through manageable digital tools, and with minor technical issues that were subsequently resolved. They described feeling safe and supported in the digital environment, fostering a sense of community and individual focus. The accessibility of home-based participation facilitated adherence, making the intervention a valuable and inclusive rehabilitation option for breast cancer survivors, particularly for those experiencing fatigue, time constraints, or challenges related to traveling.

**Conclusions:**

The digitally distributed home-based yoga intervention was experienced as an effective and accessible tool for breast cancer rehabilitation, promoting overall well-being. For these participants, it fostered resilience, empowerment, and a sense of community; this highlights its potential as a user-friendly rehabilitation option that could be integrated into cancer care to support recovery and address various patient needs.

**Trial Registration:**

ClinicalTrials.gov NCT04812652; https://clinicaltrials.gov/study/NCT04812652

**International Registered Report Identifier (IRRID):**

RR2-10.1136/bmjopen-2022-065939

## Introduction

### Background

Breast cancer is the second most prevalent cancer, with an estimated 2.3 million new cases globally in 2022, comprising 11.6% of all cancer diagnoses [1]. Advances in cancer screening, diagnostic methods, and treatment have significantly improved survival rates [2]. However, breast cancer survivors increasingly have to confront long-term effects from both the disease and its treatment, including physical, functional, emotional, and psychosocial changes that impact quality of life [3].

Cancer-related fatigue (CRF) is the most common and disruptive symptom among breast cancer survivors [4], with profound negative effects on daily functioning and quality of life [5]. CRF is defined as a distressing, persistent, subjective sense of physical, emotional, or cognitive exhaustion, or a combination of these, related to cancer or its treatment, disproportionate to recent activity and significantly interfering with normal functioning [6]. It can result from treatment-related factors and the symptom burden of the disease, as well as its psychological burden [7]. Approximately 1 in 4 breast cancer survivors suffer from severe CRF [8], which is associated with higher levels of depression, sleep disturbance, and pain [7].

Physical activity is a key recommendation for mitigating the adverse physical and psychosocial effects of cancer and its treatment. It not only reduces the risk of secondary malignancies and comorbidities but also enhances quality of life [9,10]. Regular exercise is demonstrated to be particularly effective in alleviating CRF and improving health-related quality of life both during and after breast cancer treatment [5,11-14]. However, many patients with cancer experience a decline in physical activity levels following diagnosis [15] due to various barriers, including stress [16], reduced self-confidence in their physical abilities, little confidence in the perceived benefits of physical activity, and a lack of prior experience with structured physical activity [17].

Breast cancer rehabilitation requires a comprehensive and multidimensional approach to optimize health-promoting strategies [3]. Yoga is increasingly recognized as a complementary intervention to mitigate cancer-related symptoms and improve well-being [18,19]. It is a low-cost and safe practice, recommended to be integrated into standard cancer care [18]. Depending on treatment status, yoga may be more effective than traditional aerobic or strength exercises in preventing or reducing CRF [13,14]. Additionally, yoga has emerged as a popular rehabilitation option among patients with breast cancer [20].

Digital interventions such as telerehabilitation are an emerging avenue for integration into standard cancer rehabilitation, supporting a shift toward person-centered care while offering health-economic benefits [21]. Telerehabilitation refers to the delivery of rehabilitation services through digital communication technologies and encompasses a wide range of clinical activities, including interventions such as exercise programs, education, and self-management support [22]. Delivery may occur synchronously, in real time, or asynchronously, with a time delay between transmission and reception. A variety of technological formats are used, including web-based systems, mobile apps, and different combinations of digital technologies [23].

Digital physical activity interventions have been demonstrated to improve activity levels, physical function, and health-related quality of life [24]. Despite their potential, digitally distributed yoga interventions remain underexplored in the breast cancer setting, and existing studies vary in quality [25-28]. There is a pressing need for high-quality digitally distributed yoga interventions in order to expand treatment options and improve accessibility, particularly for patients with breast cancer at higher infection risk, for those living in remote areas with limited access to organized rehabilitation programs, and for further research on such interventions.

The randomized controlled trial, Digital Yoga Intervention in Cancer Rehabilitation (DigiYoga CaRe), investigates the efficacy of a 12-week digitally distributed yoga intervention to alleviate CRF and stress and to enhance health-related quality of life and physical activity levels [29]. While studies using quantitative methods provide valuable insights, a deeper understanding of participants’ experiences requires qualitative exploration, especially in the context of novel treatments. Given the innovative nature of this intervention within breast cancer rehabilitation, it is essential to explore participants’ experiences in order to inform future implementations and optimize the integration of this intervention into clinical practice.

### Aim

The aim of this study was to explore participants’ experiences of engaging in a home-based, digitally distributed yoga intervention, which included both live-streamed and prerecorded instructor-led yoga sessions, as part of their breast cancer rehabilitation.

## Methods

### Design

This study used a qualitative design using conventional content analysis with an inductive approach, as described by Hsieh and Shannon [30]. Qualitative interviews provide deep insights into the interviewees’ feelings, thoughts, and intentions [31]. Moreover, the qualitative analysis process considers both the interviewees’ perspectives and contextual factors when categorizing the interview transcripts into related themes by identifying similarities, differences, patterns, and associations at both explicit and implicit levels [30,32]. Based on these considerations, this study design was deemed appropriate. The COREQ (Consolidated Criteria for Reporting Qualitative Research) checklist was followed to ensure explicit and comprehensive reporting [33].

### The DigiYoga CaRe Trial

This study is part of the larger randomized controlled multicenter trial, DigiYoga CaRe [29], which included 228 participants. Women diagnosed with breast cancer who had undergone surgery were recruited between October 2021 and March 2023 from 5 Swedish hospitals, as well as through self-referrals. Data were collected at multiple follow-ups using questionnaires, physical activity measures, blood samples, and clinical records. The trial is ongoing, with participants scheduled for follow-up at 48 months; results are anticipated after this period.

Those eligible for inclusion in the trial were adult women who were literate in Swedish, had undergone breast cancer surgery with curative intent, and had no signs of metastatic disease. Recruitment took place within 60 days after the surgery, following the regular postoperative follow-up visit, but before the start of any planned chemotherapy. Exclusion criteria included having received neoadjuvant treatment or having unstable cardiovascular disease. Women with physical conditions that prevented participating in yoga, or unstable medical conditions that could be aggravated by yoga, were also excluded. Participants were randomized through a web-based digital portal either to a 12-week digitally distributed, home-based yoga intervention in addition to standard care (intervention group) or to standard care alone (control group).

To minimize design ambiguities, the yoga intervention was developed following Sherman’s recommendations [34] and is detailed in the DigiYoga CaRe study protocol [29]. The intervention included yoga postures and movements tailored to breast cancer survivors, concluding with relaxation and reflection.

The intervention group practiced yoga twice weekly at home: each week, there was 1 live-streamed class and 1 prerecorded self-paced practice video. Participants entered the intervention at different time points, creating a dynamic and diverse group composition. Each live-streamed class was led by an experienced yoga instructor. Two instructors led the classes independently, each responsible for 1 weekly session held consistently on the same weekday and time throughout the intervention. At the beginning of the intervention, participants chose which of the 2 weekdays they preferred to attend. Before the intervention, the yoga instructors completed a 1-day course led by the research team, covering the following topics: breast cancer and treatments; study objectives and design; yoga theory and practice; and modification and digital intervention strategies.

The yoga sessions followed standardized programs designed to (1) increase energy levels, (2) reduce fatigue, (3) increase calmness and focus, and (4) improve acceptance and coping strategies. Participants were provided with two different 6-week programs during the 12-week intervention. Each 60-minute session included 10 minutes of relaxation and, in the live-streamed classes, a 5-minute voluntary reflection period.

The instructor opened the digital meeting room approximately 15-30 minutes before each live-streamed class, primarily to allow participants to check the technology and ask questions, but also to provide an opportunity for informal group connection. Participants were also given the possibility to stay for a few minutes after the reflection period to ask questions. Instructors offered modifications based on individual needs, and the prerecorded videos included multiple adaptation levels.

The participants were expected to practice a yoga program individually using the prerecorded videos once a week in addition to the live-streamed yoga class. The videos followed the same sequence and content as the live-streamed classes. For each 6-week program, both instructors had recorded the same yoga sequence, resulting in 2 instructor versions of each class. Participants could choose freely from a library of 3 prerecorded videos: one guided by each of the two instructors, both offering modification options, and a third simplified version without modification instructions. In total, 6 videos were available across the 2 programs. Adherence to the live-streamed yoga classes was noted by the instructors, while engagement with the prerecorded videos was self-reported using a web-based questionnaire.

Participants were expected to use their own computer, tablet, or mobile phone. The live-streamed yoga classes were conducted via the Zoom platform, with end-to-end encryption. Written basic information, including instructions on how to connect to the live-streamed yoga class, was sent by email. Participants were offered technical test sessions once or twice weekly before they started the intervention. Personalized support was also offered, which was a prebooked, 15-minute individual training to use the digital technology. For the yoga sessions, participants were encouraged to use a soft mat, blankets, pillows, a large book (or a yoga block), and a chair if needed. After completing the 12-week intervention, participants received an email encouraging them to continue practicing yoga with the prerecorded videos provided in the trial.

### Sample

In this qualitative study, 20 women from the intervention group were purposively selected for individual interviews to ensure a diverse and rich dataset [35], a variation in age, and a proportional ratio of participants recruited to the DigiYoga CaRe trial through hospital referrals and via self-referrals through social media. The target sample of 20 participants was considered sufficient to capture diverse and rich experiences, aligning with Hsieh and Shannon’s suggestion that approximately 20 participants can provide adequate variation [30].

After completing the 12-week intervention, the selected women were contacted by email, telephone, or both and invited to participate in an interview. The interviewees were selected by researchers who had no previous personal contact with them or insight into the live-streamed yoga class.

### Data Collection

A semistructured interview guide containing 10 open-ended questions was developed by the research group. The first 2 interviews were conducted by PWTF, a registered nurse, and were considered pilot interviews. These pilot interviews underwent careful review and discussion by the research team, and the original interview guide was supplemented with 2 additional questions concerning participants’ bodily and mental experiences during and after the yoga class. The original questions explored the interviewees’ experiences of the home-based yoga intervention, reflections on the digital format, the structure of home-based training, and how the intervention influenced their everyday lives. The final version of the interview guide, including all 12 questions, is presented in Textbox 1.

**Interview guide.**

- What was it like to participate in digital yoga sessions?
- What was it like to connect on the internet to join the yoga group classes?
- What was it like to do yoga with the prerecorded video?
- How did you experience being at home practicing yoga and at the same time being part of a group of women operated for breast cancer also doing yoga at home?
- How did you experience the group?
- How did you feel in your body during and after the yoga session?
- How did you feel mentally during and after the yoga session?
- Can you tell me about what has been less good for you?
- Can you tell me about what has been good for you?
- How would you want an online class to work?
- Is there anything you want to add before we end the interview?
- Do you have any good advice for others who want to start doing yoga online?

All interviewees were encouraged to speak freely and elaborate on their answers, and follow-up questions were used to gain deeper insights [30,36]. Neither interviewer had prior experience with digitally distributed live-streamed or video-recorded yoga sessions or with yoga as a rehabilitation modality in cancer care. Additionally, the interviewers had no therapeutic relationship with the participants.

The selection of interviewees started in June 2022 and continued until 20 individuals had been included, in June 2023. The first 2 interviews conducted in June 2022 as pilot interviews were included in the analysis as only minor adjustments were required. The remaining 18 interviews were conducted by MBN, a registered physiotherapist, between October 2022 and June 2023.

The interviews were conducted within 1 month after the completion of the 12-week yoga intervention, by online video call, by telephone, or in person, according to the interviewee’s preference. Overall, 17 interviews were conducted by online video call using the digital patient visits system used in Region Örebro County, while 3 interviews were conducted by telephone. One online video interview was repeated due to failure of the audio recording. The interviews lasted between 8 and 33 minutes, with a mean duration of 21 minutes.

All interviews were audio-recorded and pseudonymized. The audio recordings were transcribed verbatim, 2 by MBN and the remaining 18 by experienced transcribers. The accuracy of the transcripts was confirmed by MBN by concurrently reading them and listening to the corresponding recorded interviews.

### Data Analysis

The interviews were analyzed using qualitative content analysis with a conventional, inductive approach, as described by Hsieh and Shannon, to capture direct information from the interviewees without imposing predefined categories or theoretical perspectives [30]. The results thus reflect the interviewees’ unique perspectives and are grounded in the actual data.

The interview transcripts were imported into NVivo 14 (QSR International), which facilitated data management and the analysis process. The analysis began with two of the authors (MBN and AD) independently reading all transcripts several times to gain a comprehensive understanding of the material [30]. Throughout the analysis, transcripts were repeatedly reviewed to gain an overall understanding of the data, with continuous comparisons between individual data segments and the full interview content. MBN took initial notes capturing first impressions, ideas, and preliminary analytic thoughts. Next, meaning units relevant to the study aim were identified and coded. Each meaning unit was condensed and assigned a code, closely aligned with the original text.

MBN and AD continuously discussed the identified codes and potential categories and sorted them into meaningful clusters, forming the basis for developing subcategories. All authors were then engaged in the process of discussing and sharing their impressions of the identified codes and merging subcategories. Discrepancies in coding and subcategories were discussed until agreement was reached by the authors. To enhance trustworthiness, reflexivity was maintained through ongoing discussions among authors representing different professional and theoretical perspectives during the analysis. The analysis proceeded until the authors agreed that the data provided a comprehensive understanding of the participants’ experiences and adequately reflected the identified categories.

MBN systematically structured the subcategories into fewer, overarching main categories, continuously maintaining a dialogue with AD throughout this process. Through iterative analysis and abstraction, 3 main categories emerged. Authors who had not read all the transcripts contributed additional perspectives, helping to refine conceptual clarity and ensure a nuanced representation of interviewee narratives within the categories. An example of the analysis process is illustrated in Textbox 2.

Representative quotations were selected by MBN and discussed among the authors, with final quotations chosen by consensus to illustrate each subcategory effectively.

**Example of the analysis process.**

**Meaning unit**

- Just that it’s not scary, that even if you think “I'm so clumsy and awkward,” yes, but you can do it as best you can, everything is, you don't have to be like an athlete, everything is okay. And yes, but it was also nice to be at home.
- It's incredibly valuable to have access to this at home and not have to leave home, not have to go out and, for me, maybe it’s an effort to get to somewhere, then it takes time, it takes energy, it takes, you know. Then it’s fantastic to have it at home, then it gets done. I think that’s a great advantage to have it, to have it available where you live.

**Code**

- Not scary even if clumsy, awkward, do the best you can, everything is okay, nice to be at home
- Incredibly valuable to not have to leave home, an effort to get out, it takes time, it takes energy, gets done at home.

**Subcategory**

- Feeling safe and supported in the digital environment
- Promoting accessibility and facilitating participation

**Category**

- A safe and user-friendly rehabilitation option

### Ethical Considerations

This study, as part of the DigiYoga CaRe trial, was conducted in accordance with the Declaration of Helsinki [37] and approved by the regional ethical board in Lund, Sweden (approval numbers 2020-06219, 2022-00956-02, and 2021-05994-02).

Participants received written and oral information detailing the voluntary nature of participation, including the right to withdraw from the study at any time without providing a reason. All participants provided both written and oral informed consent prior to participation. No compensation was provided for participants in the intervention group. To ensure confidentiality, personal data were coded and deidentified prior to analysis. Study materials for the randomized controlled trial were managed in the web-based system Greenlight Guru Clinical, while audio recordings and transcribed interviews were securely stored in Region Örebro County’s FoU Drive. Both systems comply with regulations for the secure handling of sensitive research data, and access was restricted to authorized study staff only.

## Results

### Sample

Of the 24 women contacted, 2 women declined to participate due to time constraints, and 2 others could not be reached by email or telephone. A total of 20 women were thus included in the study. At baseline, self-reported data on participants’ demographic characteristics and medical background were collected and entered into the web-based system Greenlight Guru Clinical, and the trial participation rate was documented during the intervention. The urban-rural classification for each participant was based on their residential address, and the classification by urban size was based on data from the SCB (Swedish Statistics Agency) 2023 [38] (Table 1).

**Table 1:** Characteristics of the interviewees (N=20).

Characteristics			Values
Age in years, mean (range)			56 (37-80)
**Community type, n (%)**			
	Urban area, >200,000 inhabitants	3 (15)	
	Suburban area, >40,000 inhabitants	5 (25)	
	Suburban area, >15,000 inhabitants	6 (30)	
	Rural area, <15,000 inhabitants	6 (30)	
**Type of surgery,** **n (%)**			
	Mastectomy	9 (45)	
	Lumpectomy	11 (55)	
**Type of treatment, n (%)**			
	Chemotherapy alone	4 (20)	
	Antihormonal treatment alone	13 (65)	
	Chemotherapy + antihormonal treatment	2 (10)	
	Surgery alone or with radiation	1 (5)	
**Recruitment method, n (%)**			
	Hospital	7 (35)	
	Self-referral	13 (65)	
**Live-streamed yoga participation rate, n (%)**			
	All (12)	3 (15)	
	Most (6-11)	17 (85)	
	A few (1-5)	0 (0)	
**Prerecorded video participation rate^a^, n (%)**			
	All (12)	9 (47)	
	Most (6-11)	8 (42)	
	A few (1-5)	1 (5)	
	None (0)	1 (5)	

^a^Data missing for 1 interviewee.

### Emerging Categories and Subcategories

#### Overview

Analysis of the data from the 20 interviews yields three categories related to the interviewees’ experiences of participation in DigiYoga CaRe: (1) actively managing illness and side effects of cancer treatment, (2) building resilience and reclaiming empowerment, and (3) a safe and user-friendly rehabilitation option. Each category is abstracted from 3 to 4 subcategories (Textbox 3).

The categories and subcategories are described below, illustrated by quotations from the interviewees.

**Categories and subcategories.**

**Actively managing illness and side effects of cancer treatment**

- Mental recovery and relaxation
- Physical function and activity
- Self-care practices and routines

**Building resilience and reclaiming empowerment**

- Body awareness and regaining trust in one’s body
- Self-confidence and hope for recovery
- Shifting focus toward progress and well-being
- Coping strategies for daily life

**A safe and user-friendly rehabilitation option**

- Managing digital tools and technical challenges
- Feeling safe and supported in the digital environment
- Sense of community and individual focus
- Promoting accessibility and facilitating participation

#### Actively Managing Illness and Side Effects of Cancer Treatment

##### Category Overview

This category encompasses the yoga intervention as a proactive approach to the recovery of mental functions and relaxation, and the restoration of overall well-being, while also enhancing physical function and activity levels. Several interviewees described how mental and physical recovery were closely intertwined throughout their yoga sessions. They emphasized that addressing both aspects simultaneously was essential in managing their illness and the side effects of the cancer treatment. Additionally, the digitally distributed yoga intervention helped them to develop structured self-care practices and establish consistent daily routines.

##### Mental Recovery and Relaxation

The interviewees described participation in home-based yoga as facilitating mental recovery, relaxation, and the restoration of well-being. The interviewees reported that cancer and its treatment were challenging experiences, and they found that practicing yoga played a supportive role in the mental healing process. They described cancer and surgery as sources of significant inner stress, with yoga helping to foster a sense of calm and contributing to the restoration of mental well-being. They emphasized the meditative aspects of yoga, which involved focusing on self-care and processing emotions related to their cancer journey, as beneficial for their mental recovery. By redirecting attention away from pain, limitations, and negative thoughts, they experienced a more positive and present-focused mindset. One interviewee said:

It has helped in the healing process and in, well, the healing process of the mind as well, if you’ve had bad days or been low, it has helped there too.

Interviewee 15

Several interviewees emphasized that yoga helped them relax, with their minds regaining a sense of calm after a yoga session, leading to improvements in well-being and increased energy levels. Focusing on breathing helped them to relax and disconnect from overwhelming thoughts. Breathing exercises, along with mindful engagement with the body, intentional movements, and postures, helped them to feel more relaxed and in harmony. For many, this mind-body connection was a central aspect of their overall healing process.

##### Physical Function and Activity

The interviewees described yoga as a versatile form of exercise that works the entire body, with many reporting improvements in strength, mobility, and balance as a result of their participation in the yoga intervention. They described how practicing yoga enhanced their physical function and restored vigor so that they felt stronger and more capable. Practicing yoga helped them to stretch the operated area and improve mobility in the arm on the operated side. Some interviewees mentioned stiffness and pain as side effects of cancer treatment and found that they could use yoga as an active tool to counteract these issues.

You get stiff after an operation and your body gets stiff from treatments. So that stretching and the one in yoga and like these exercises have been, have been really useful. The breathing, as I said, was super important too, nice, that, yes, no but I think the stretching, it’s probably the be-all and end-all for me that has been super positive.

Interviewee 15

Yoga was also described as an effective way to resume physical exercise after surgery and as a means to enhance overall physical activity. Most interviewees reported continuing or intending to continue yoga after the intervention, and many also started other forms of exercises and activities.

However, some interviewees faced challenges; one described difficulty practicing yoga due to pain and old injuries, which led her to discontinue yoga and choose alternative activities better suited to her needs. Another interviewee mentioned muscle strain as a potential side effect. Despite these challenges, yoga remained a valuable therapy for most of the interviewees, contributing to their physical and mental well-being.

##### Self-Care Practices and Routines

The interviewees described doing yoga at home as an effective way of structuring self-care practices and establishing daily routines. Many emphasized the value of having a set time for yoga, which helped restore structure and discipline, particularly during a period when their usual routines were disrupted by illness and sick leave. They reported that the scheduled yoga classes played a key role in promoting adherence and self-discipline, contributing to consistent involvement.

Personally, I felt some days like, “Ugh, no, I can’t do it, I don’t want to,” but I still turned on the camera or the computer, logged in, and it was so nice because I knew that if I wanted to [but] couldn’t do anything, I could just lie there and listen while she told me. But at least you were like, “Yes, but no, but I’m doing it, I’m doing what I can, what I can manage.” So it felt really good afterwards.

Interviewee 19

The interviewees emphasized that they saw the home-based, digitally distributed yoga classes as an intentional act of self-care, allowing them to prioritize their well-being. They appreciated the opportunity to dedicate time to themselves, with yoga serving as a personal moment of valued self-care.

Additionally, some also highlighted the understanding and support they received from their families and employers, in respecting moments dedicated to self-care, which facilitated their ability to consistently participate in the sessions. Several of the interviewees stated that they would continue yoga according to a structure that suits them. Some, however, said that it was easier to establish routines with the live-streamed yoga sessions and more difficult to keep to the structure with the prerecorded videos.

#### Building Resilience and Reclaiming Empowerment

##### Category Overview

This category encompasses regaining hope and a sense of control and agency. Several interviewees reported that yoga helped them improve their body awareness, rebuild trust in their bodies, and overcome barriers to movement. They also commented that yoga enhanced their mindful presence, self-confidence, and hope for recovery. By shifting their focus toward well-being, they observed progress in daily functioning. Moreover, several interviewees described how yoga supported them in developing effective coping strategies for managing the challenges of daily life. Participating in home-based yoga offered them a means to regain control over their physical and mental health, leading to increased feelings of empowerment and resilience in their recovery process.

##### Body Awareness and Regaining Trust in One’s Body

The interviewees described how practicing yoga at home contributed to improving their body awareness, regaining trust in their bodies, and overcoming barriers to movement. They commented that yoga allowed them to recognize themselves in their bodies and regain control. It also supported them in rebuilding trust in their physical abilities and feeling more confident in their movements.

It's different every time, and different in different places. But...as a whole, I feel inside myself, I feel myself in my body, so I am in, I have control over my body and control over life, I have control over myself and, like, everything in some way.

Interviewee 13

Interviewees described how practicing yoga helped them overcome barriers to movement and encouraged them to use the arm on the operated side more confidently in daily activities. Yoga allowed them to realize that it was safe to move and use that arm, even when it felt tight or painful.

With yoga, you have to use your whole body, so I discovered that I don’t have to be afraid of this, I can push on it as usual and I can, yes. I would say that, that was the biggest thing it has given me.

Interviewee 13

##### Self-Confidence and Hope for Recovery

Practicing yoga contributed to enhancing mindful presence, self-confidence, and hope for recovery. Yoga supported the interviewees in re-establishing their emotional resilience, enabling them to move forward by focusing on opportunities rather than obstacles.

In my work-life balance as it looked or has looked in this period of my life which has been quite chaotic in many ways, I have still been able to gather myself together and move on through yoga. To see opportunities instead of obstacles. So I am very happy for that.

Interviewee 13

Some interviewees valued the sense of togetherness within the online group, noting that being with others in a similar situation contributed to feeling happier and more hopeful for the future. They commented that being part of a group with shared experiences fostered a sense of belonging, which in turn brought hope for the future, improved mood, and a more positive outlook.

##### Shifting Focus Toward Progress and Well-Being

The interviewees described that participating in yoga at home contributed to shifting focus toward well-being and to observing progress in daily functioning, both physically and mentally. Through the repetition of yoga sequences over several weeks, they became more aware of their improved ability to perform movements with greater ease. For instance, they noticed an improvement in mobility of the arm on the side of the breast cancer surgery, allowing them to raise that arm fully toward the ceiling.

Several interviewees noted that, through practicing yoga, they saw progress in their mental health, along with improvements in their energy levels and cognitive functions. Through this process, they demonstrated a growing resilience, overcoming cognitive challenges and regaining their sense of normalcy. For example, one interviewee specifically highlighted how yoga helped restore her mental clarity and improve her memory.

...and you felt that it would get better, it’s just like you noticed with the yoga that you became more and more skilled after more weeks of doing it and that also made your brain keep up. So I thought after five or six times I was aware that I felt better and that my brain had come back, the forgetfulness or whatever, it started to disappear, I remembered normal things again like shopping on the way home from work and things like that.

Interviewee 16

##### Coping Strategies for Daily Life

Several interviewees described developing effective coping strategies for daily life through their new yoga skills. This included the use of breathing techniques to alleviate tension and relaxation techniques to promote sleep. Additionally, some interviewees mentioned incorporating yoga postures, such as stretching and balance exercises, into their daily routines.

I have continued with breathing exercises at certain times during the day, when I have difficulty falling asleep, or something like that, or unwinding, I come back to...my breathing exercises, so I have definitely carried with me what we have kind of done in yoga.

Interviewee 15

#### A Safe and User-Friendly Rehabilitation Option

##### Category Overview

This category incorporates the interviewees’ experiences with the digital aspects of the yoga intervention and the home-based participation. Overall, they regarded the digitally distributed yoga intervention as a safe and user-friendly rehabilitation option, offering an accessible and flexible means of participating in rehabilitation after breast cancer surgery. They commented that the home-based nature of the intervention enabled them to engage in rehabilitation at their own pace and within the comfort of their own space, ensuring convenience and safety. They considered the digital tools (live-streamed Zoom classes and remote access to prerecorded videos) manageable and intuitive, enhancing the overall experience.

##### Managing Digital Tools and Technical Challenges

The digital technology was a key aspect of the interviewees’ experiences with the yoga intervention. Most of them were familiar with the technology and found the digital tools easy to handle, with many of them describing prior experiences with similar tools in other contexts.

I kind of thought, “I’ll never be able to do this,” but it went really well, there were no problems, I followed what was written to the letter and it works, it works every time.

Interviewee 9

Despite this, some interviewees did report encountering technical challenges, particularly during their first yoga session. Common issues included connectivity, audio, or video challenges, although these issues were subsequently resolved. One interviewee described an instance with difficulty hearing the instructor, which was resolved by switching to her mobile phone, while another experienced poor audio quality, which was improved by using an additional speaker. Connectivity problems were experienced by participants and also the instructor at times, but these were brief. One interviewee described a delayed video feed, which made it challenging to follow the instructor’s movements, though this issue was temporary.

But the first time it was a bit awkward, and it was, uh, hard for me to get a good sound, and then I connected my computer to the TV and then I could get a better sound. But since then it has worked.

Interviewee 1

##### Feeling Safe and Supported in the Digital Environment

The interviewees described feeling safe in the online group with others in the same situation. Most of them felt seen and included and described a sense of reassurance provided by the yoga instructors and a supportive atmosphere in the group. The interviewees emphasized being able to engage in the sessions at their own pace and according to their individual capabilities. Some pointed out that there was no pressure to perform, which contributed to a sense of ease throughout the sessions. This approach, focused on their well-being, allowed them to feel safe, relaxed, and engaged in the yoga sessions.

I haven’t had to worry about it being difficult or anything, the classes have been designed in such a way that I think I’ve felt relaxed the whole time. No pressure to perform.

Interviewee 20

The interviewees appreciated the variation in the intervention format, which included both live-streamed classes and prerecorded videos. This provided greater flexibility, allowing for individual choice of instructors and different programs tailored to personal preferences. Most of the interviewees favored the live-streamed classes, describing them in positive terms regarding the instructors and the selection and design of exercises. They appreciated the ability to participate regardless of their condition on the day, commenting that the exercises were adapted to various levels of difficulty and were simple and easy to follow. Several interviewees highlighted the benefit of exercises being offered with varying difficulty levels.

It was also really good that they showed different, that is, the same movements but with the possibility of sitting on the chair or being on the floor (...), that there were different levels of difficulty.

Interviewee 9

Some interviewees reported the benefits of the prerecorded videos, as these provided greater autonomy over their yoga practice and the flexibility to choose when to engage in yoga. One interviewee highlighted and appreciated the ability to pause the video, reflect on her movements, and ensure she was properly aligned with the instructions.

##### Sense of Community and Individual Focus

This subcategory concerned the live-streamed yoga classes. The group dynamic created a supportive environment, allowing participants to engage with the yoga exercises at their own pace while still feeling part of a collective experience. The interviewees described how the personal and welcoming atmosphere, with an individualized welcome from the instructor, fostered a sense of connection. They also appreciated the shared understanding within the group, as everyone had similar health challenges. This shared experience created a safe space where participants could share challenges without the need for detailed explanations.

If nothing else, it’s nice to know that there are many others who are affected. Then there’s knowing that there is a fragility that is hard to describe to someone who hasn’t experienced it. Just that in itself I think is reassuring, it’s like no one needs to explain themselves or someone who says “I can’t manage much today” or something like that, it’s nothing strange, it’s an accepting atmosphere, so to say.

Interviewee 13

Seeing other women in a similar situation, such as noticing the absence of hair, along with the presence of personal items and pets in their fellow participants’ homes, further fostered a sense of shared space and camaraderie within the group. This created a feeling of connection with others who were facing similar challenges.

While some interviewees highlighted the positive aspects of the group environment, others expressed that the limited opportunities for interaction outside of the instructor’s guidance prevented deeper connections. The sense of anonymity hindered the development of a more cohesive and supportive group dynamic.

It still becomes very individual when we’re all lying there. And you don’t look at anyone else, you just concentrate on yourself. Uh, and then it’s kind of a short conclusion after we’ve had our session, how we’ve experienced it, but I don’t think there’s that group feeling, there’s no real group feeling, at least not for me.

Interviewee 1

Despite the overall positive experience within the yoga group, some interviewees expressed a desire for more interaction with other participants. Suggestions included creating a closed group with a fixed membership to strengthen the sense of community and incorporating discussions about the shared experience of breast cancer to foster deeper connections. However, one interviewee particularly expressed a preference for avoiding a focus on the illness or comparing experiences, instead valuing an emphasis on the yoga itself.

##### Promoting Accessibility and Facilitating Participation

Several interviewees reported that attending yoga from home reduced the effort and energy required, making it more feasible for individuals facing challenges such as CRF, adverse weather conditions, and time constraints.

I think yes, but the thing about it being online, because when you’re on chemotherapy, when you’re this tired, you can’t manage to go anywhere, it’s not possible and then it’s really, really nice to be able to do it at home, it is.

Interviewee 14

The interviewees also noted that yoga at home offered convenience and time-saving benefits, allowing them to engage in the yoga sessions without the need to travel. This approach further enhanced accessibility, enabling participation regardless of geographic location, thus fostering greater inclusivity in the rehabilitation. Moreover, the time saved by eliminating the need for travel allowed participants to spend more quality time with their families.

It has taken, it sounds terrible, but it has taken less time from me, I’ve used, yes I have full value for my hour simply, without having to take, to go away, because that takes even longer and it...the children have to be transported and so on in the evenings, so it is a...life you live, that’s how it is. So it has been only positive, I would say.

Interviewee 15

## Discussion

### Principal Results

This interview study explored women’s experiences with a digitally distributed home-based yoga intervention as part of their breast cancer rehabilitation. The main findings revealed that they viewed the yoga intervention as an effective, accessible, and safe tool for managing illness and treatment side effects. They reported that yoga improved their overall well-being and enhanced their resilience and empowerment, while the live-streamed group yoga classes in particular gave them a sense of community with their fellow participants and reassurance from the yoga instructors.

### Comparison With Prior Work

Our finding that digitally distributed, home-based yoga is experienced as an active way of managing illness and side effects of cancer treatment aligns with previous research [13,18,19,39-41], which has demonstrated the contribution of yoga in improving both physical and mental health. The interviewees in our study reported improvements in mental recovery, relaxation, and energy levels through home-based yoga. These benefits, particularly in stress reduction and recovery, are well established in the literature [18,40,42-45]. The interviewees also found body awareness and breathing techniques effective for relaxation, which aligns with previous research highlighting the positive effects of such practices on psychological well-being [46], as well as their ability to reduce depression, anxiety, and CRF in patients with cancer [47-49]. Consistent with these findings, studies of patient experiences with online mind-body programs (which can include yoga, meditation, tai chi, cognitive skills, lifestyle interventions, and positive psychology) have been shown to enhance psychological coping, to support adherence to health behaviors, and to increase social connections among patients with cancer [50,51]. Moreover, our study reveals additional benefits, with digitally distributed yoga being perceived as a safe and user-friendly rehabilitation option.

The interviewees in our study reported improvements in physical function and activity level through yoga, including mobility, reduced stiffness and pain, and improved strength, flexibility, and balance. These findings align with previous research on the reasons patients with breast cancer engage in yoga after diagnosis [41]. Many interviewees expressed a desire to continue practicing yoga beyond the intervention, viewing it as a means to maintain or further increase their physical activity. Previous research indicates that learning yoga encourages independent practice, improves quality of life, and promotes physical activity [52]. This is particularly important, as physical activity and exercise have been shown to positively impact physical function, pain, CRF, and quality of life following breast cancer treatment [43].

Joint and muscle pain, which are among the most frequently reported side effects of yoga when these occur [41], were also noted by interviewees in our study. Physical side effects in general are recognized as significant barriers to exercise during and after cancer treatment [53]. This highlights the importance of tailoring exercise programs to meet individual needs, as emphasized by previous research [54]. Given that various nonpharmacological interventions can offer similar benefits, it is essential for health care professionals to consider individual patient preferences when planning optimal rehabilitation [14]. This also serves as a reminder that yoga may not be suitable for all patients with breast cancer, as some may not experience the same benefits or may prefer alternative forms of exercise. Our findings align with previous research that underscores the variability in patients’ interests [20] and needs [54] in cancer rehabilitation. This further emphasizes the importance of identifying each patient’s specific needs to provide a diverse range of rehabilitation options for cancer survivors.

This study highlights the role of structured yoga sessions in helping participants establish routines and self-care habits. Some of our interviewees emphasized the importance of support from family or employers in respecting time dedicated to self-care. This underscores the critical role of social support in sustaining health behaviors in general, aligning with research on the impact of social support specifically on the health behaviors of individuals living with or beyond cancer [55]. The live-streamed classes in our yoga intervention were particularly effective in maintaining adherence, while some interviewees found it more challenging to consistently follow the schedule for prerecorded videos. This suggests that real-time engagement and social interactions may enhance commitment and routine building, as the live-streamed classes provided a more structured environment and an immediate sense of community.

In our study, the interviewees described how regaining trust in their bodies was a key to building resilience and reclaiming empowerment. By overcoming physical limitations, interviewees were able to engage in activities that supported their recovery and improved their quality of life. This was accompanied by increased resilience, with interviewees reporting enhanced self-confidence and hope for recovery. Our findings suggest that fostering self-confidence and hope enables individuals to face challenges with a positive mindset, reinforcing their belief in overcoming obstacles and achieving well-being. A shift in focus can help reduce feelings of helplessness and empower individuals to take an active role in their physical and emotional well-being. By focusing on the present moment, participants could move beyond pain and limitations, concentrating on the body’s strengths while promoting resilience, self-compassion, and acceptance.

As described in our study, resilience—the ability to bounce back from stress [56]—is particularly important during the recovery phase following breast cancer surgery. Research suggests that resilience-enhancing strategies, self-management training, and social support in the early stages after breast cancer diagnosis can improve both quality of life and personal resilience [57]. Our study further highlights the role of empowerment in postsurgical recovery. As defined by the World Health Organization, empowerment involves the individual’s ability to make decisions and have control over their personal health [58]. In the context of promoting self-management behaviors among posttreatment patients with cancer, empowerment is further conceptualized as a process of personal growth, characterized by fortitude and strength [59]. This process occurs when patients with cancer gain control over the impact of their illness, no longer perceiving cancer as a threat, as described by interviewees in our study. Empowerment was further reinforced as they observed tangible progress in their health and daily functioning. Improvements in mobility, energy levels, and the ability to perform daily tasks cultivated a sense of achievement, which motivated continued commitment to self-care practices.

Our findings suggest that developing effective coping strategies for daily life plays an important role in both resilience and empowerment, providing individuals with the tools to take control of their long-term health and well-being. The interviewees described how, by learning to manage the challenges of posttreatment recovery, they were able to further apply these strategies in everyday life, such as dealing with stressful situations or sleep disturbances. We believe these factors collectively contribute to empowering patients with cancer to engage in self-care, thus enhancing their control over their health. In turn, this aligns with the broader concept of empowerment through self-management behaviors, helping to build resilience and personal strength throughout the recovery process.

Most interviewees in this study found the digital tools used for the yoga intervention (live-streamed Zoom sessions and remote access to prerecorded videos) easy to manage, largely due to prior experience with digital platforms in their work or daily life. This familiarity facilitated smooth adoption of our technology-based health intervention. While a few interviewees had encountered minor technical challenges, such as connectivity, audio, or video problems during their first live session, these were quickly resolved, indicating that such difficulties do not pose significant barriers to implementing digital health interventions. These findings align with the increasing use of a variety of digital health tools in cancer care, which provide new opportunities for patient management, enhancing the delivery of safe and effective cancer care [60].

In this study, several interviewees reported feeling a sense of safety, reassurance, and inclusion within the digital yoga group. They appreciated the supportive environment cultivated by both the instructors and fellow participants, which fostered a sense of belonging. This finding aligns with previous research on virtual mind-body programs for patients with cancer, which has shown that such programs enable patients to connect and engage socially [50].

The digital environment allowed participants to engage comfortably regardless of their physical or emotional state. This sense of acceptance, of a sometimes lower performance level, was further enhanced by the instructors’ ability to accommodate different skill levels and provide modifications to meet individual needs, fostering a nonjudgmental environment where participants could progress at their own pace. Yoga is most beneficial when tailored to an individual’s needs and abilities, and practiced under the guidance of an experienced instructor with medical knowledge to ensure safety and effectiveness [61]. Therefore, it is essential that yoga interventions for women with breast cancer are appropriately adapted to address their specific needs. In our study, the sessions were led by experienced instructors who had received specialized education from the research team on yoga for patients with breast cancer, which may have contributed to the sense of safety reported by interviewees.

Disease and treatment barriers such as feeling sick, CRF, and pain are frequently reported by women with breast cancer undergoing chemotherapy, often preventing them from participating in conventional gym-based exercises [62]. In our study, the interviewees described digitally distributed home-based yoga as a motivation to exercise despite the side effects of the illness and the challenging treatment. They appreciated the flexibility to participate regardless of their condition on the day of the session, with the option to modify intensity, shorten session duration, and adjust exercises according to personal preference. Another common barrier to exercise for women with breast cancer is being away on vacation [62], but the ability to participate remotely, regardless of geographic location, can make it easier for them to engage.

We found that interviewees appreciated the variation in the yoga format, with both live-streamed classes and prerecorded videos, offering greater flexibility. The flexibility of the digital format, including live-streamed classes that provided real-time interaction and immediate instructor support, and prerecorded videos that allowed participants to engage at their convenience and pause when necessary to reflect on their movements, was highly valued. However, most interviewees preferred engaging in the live-streamed classes, a preference supported by previous research showing that patients with cancer value the flexibility of virtual formats while also appreciating the social support of in-person yoga [63].

Previous studies suggest that telerehabilitation in integrative oncology treatments is both feasible and appealing to patients, with high utilization and satisfaction rates [64,65]. This highlights the significant potential of digital delivery in improving patient access to cancer rehabilitation. Digitally distributed, home-based yoga enables individuals to participate regardless of their geographic location, promoting a more equitable approach to rehabilitation.

### Strengths and Limitations

It is a strength of this study that the interviewers had no prior relationship with the interviewees, which likely encouraged the interviewees to speak freely about their experiences and facilitated objective and unbiased conversations. The diversity of the sample, in terms of age, community type, and type of surgery, also strengthens the study, as it contributes to a broader variation of experiences with this yoga intervention. To ensure trustworthiness, the analysis process was discussed among all authors until they reached consistency and adequacy regarding the subcategories, main categories, and interpretation.

One limitation of the study is that the interviews were conducted through online video or telephone calls and audio-recorded only, which constrained our ability to consider nonverbal cues in our communication. The duration of interviews varied considerably (8-33 minutes), and it is possible that the remote format may have led to shorter or less detailed responses than would have been obtained in in-person interviews, potentially resulting in missed information. While in-person interviews would have provided richer interaction, logistical constraints, such as the long distances between interviewers and interviewees, would have resulted in increased time and costs, which made in-person interviews unfeasible.

Additionally, 2 pilot interviews were included in the analysis despite the fact that 2 new questions were added to the interview guide as part of the analysis of the pilot data. However, the research team considered that this did not detract from the material emerging from the pilot data; the 18 interviews conducted after these adjustments provided rich material that confirmed the pilot results, and the added questions allowed new insights to emerge. A flexible approach is commonly recommended in qualitative content analysis to capture detailed responses and achieve data saturation [31].

Finally, it is important to note that the yoga intervention in this study was delivered in a controlled research setting with self-selected participants, which may affect the transferability of our findings, as those who chose to participate in the trial may differ from the broader population.

### Conclusions

Women who underwent breast cancer surgery reported that they developed resilience and reclaimed empowerment through participation in a digitally distributed home-based yoga intervention. Our interviewees described the intervention as effective, safe, and accessible, helping them manage both the physical and mental side effects of cancer treatment. From a broader perspective, this study underscores the potential benefits of integrating nonpharmacological, movement-based interventions into health care services. Such approaches can effectively address the diverse needs and rehabilitation preferences among breast cancer survivors.

Future studies could explore instructors’ experiences with delivering digital live-streamed yoga for breast cancer survivors, focusing on the challenges and strategies to enhance digital interventions. Comparative research between in-person and digitally distributed yoga classes could offer valuable insights into their impact on the participants’ well-being and satisfaction, helping to optimize rehabilitation strategies. Furthermore, exploring digitally distributed yoga for other cancer types, such as prostate cancer, could expand its applicability and effectiveness across different patient populations.

## Abbreviations

COREQ: Consolidated Criteria for Reporting Qualitative Research
CRF: cancer-related fatigue
DigiYoga CaRe: Digital Yoga Intervention in Cancer Rehabilitation
SCB: Swedish Statistics Agency

## Data Availability

The datasets generated or analyzed during this study are not publicly available due to privacy and ethical restrictions but can be obtained from the corresponding author upon reasonable request.
